# Prednisolone for COPD exacerbations: time for a rethink

**DOI:** 10.1183/23120541.00464-2023

**Published:** 2023-09-11

**Authors:** Sanjay Ramakrishnan

**Affiliations:** 1 Oxford NIHR Biomedical Research Centre and Nuffield Department of Medicine, University of Oxford, Oxford, UK; 2 School of Medical and Health Sciences, Edith Cowan University, Joondalup, Australia

## Abstract

Treatments for COPD exacerbations have not changed in the last 30 years. Despite fewer than 800 patients ever having been enrolled in placebo-controlled trials [1], systemic corticosteroids have become the main treatment of COPD exacerbations. Most of the trials were performed prior to the widespread use of inhaled corticosteroids. We know our treatment is ineffective. 28% of patients who are treated for a COPD exacerbation require re-treatment within a month [1]. We also accept a lot of harm for this marginal benefit. A meta-analysis of placebo-controlled trials concluded that prescribing prednisolone for COPD exacerbations caused more harm than benefit [1].

## Tweetable abstract

**Prednisolone given universally for COPD exacerbations causes harm without any benefit. Patients deserve blood eosinophil-guided prednisolone treatment for COPD exacerbations.** https://bit.ly/3pR2BSY

Treatments for COPD exacerbations have not changed in the last 30 years. Despite fewer than 800 patients ever having been enrolled in placebo-controlled trials [1], systemic corticosteroids have become the main treatment of COPD exacerbations. Most of the trials were performed prior to the widespread use of inhaled corticosteroids. We know our treatment is ineffective. 28% of patients who are treated for a COPD exacerbation require re-treatment within a month [1]. We also accept a lot of harm for this marginal benefit. A meta-analysis of placebo-controlled trials concluded that prescribing prednisolone for COPD exacerbations caused more harm than benefit [1]. For systemic corticosteroids to prevent one treatment failure, 10 patients had to be treated. Meanwhile, only five patients needed to be treated to cause harm. Disappointingly, pulmonary-indicated prescriptions of prednisolone now account for most of the prescriptions of prednisolone [2]. Unlike our colleagues in gastroenterology and rheumatology, we have not managed any meaningful reductions in prednisolone prescriptions in the last decade. The big question remains: how effective are systemic corticosteroids in the treatment of COPD exacerbations, particularly in primary care?

The BECOMEG study, now published in *ERJ Open Research*, tried to answer this important question. Thebault *et al.* [3] set out to recruit patients presenting to general practitioners in France with symptoms consistent with a COPD exacerbation. The authors had an ambitious target to randomise 1014 participants when the study commenced. They later revised the target down to 404 patients. Unfortunately, only 175 patients were randomised between February 2015 and May 2017. Remarkably, this is still the second largest placebo-controlled trial of prednisolone ever conducted in primary care. The difficulty the authors had recruiting participants, despite the frequency of primary care-treated COPD exacerbations, underlies the difficulty of re-evaluating entrenched interventions. Recent trials have used repeat randomisations of recruited participants to improve recruitment rates [4, 5].

Despite not reaching the prespecified sample size, the findings from the BECOMEG trial are stark. At best, prednisolone treatment failed in 42% of patients within 8 weeks after a course of treatment. Most disappointingly, placebo treatment was just as good as prednisolone therapy. Importantly, these treatment failures are not benign. A third of patients treated for a COPD exacerbation needed unplanned urgent assessment in primary care or at an emergency department. Our best treatment, prednisolone, is unhelpful and does not reduce expensive healthcare utilisation.

To make matters worse, systemic corticosteroids are one of the most harmful treatments physicians prescribe [6]. The BECOMEG investigators use an innovative analysis, Quality-Adjusted Time Without Symptoms and Toxicity (Q-TWIST), to quantify the significant harms we cause our patients by prescribing prednisolone for COPD exacerbations. This analysis is often used in oncology to assess the value of chemotherapy despite the side-effects [7]. This is exactly how we should think about prednisolone in COPD exacerbations. To do this, the authors assumed that an exacerbation lasts 8 days and weighted different adverse events that occurred in the study. This is an arbitrary simplification, not in keeping with expert consensus [8], but an important first step. The Q-TWIST analysis showed that patients treated with placebo had more exacerbation-free days than patients treated with prednisolone. There was, however, no difference in days spent with toxicity, or in time without symptoms or toxicity. Thus, unselected prednisolone for all COPD exacerbations causes the same amount of harm as placebo and fewer days without symptoms.

Where next? Three separate randomised trials [4, 5, 9] have now shown that there is a clear biomarker, the blood eosinophil count, to predict the patients who need to be treated with prednisolone. A point-of-care blood eosinophil count-guided model has been shown to be feasible and safe in primary care, while reducing prednisolone prescriptions by 30%. Prednisolone does have a role in COPD exacerbations, but only in eosinophil-high exacerbations. Placebo was better than prednisolone when treating patients with low blood eosinophils at the time of COPD exacerbation [4, 5]. Eosinophil count-guided prednisolone therapy is being tested again in a large hospital-based placebo-controlled trial in France (eo-Drive study, www.clinicaltrials.gov identifier number NCT04234360), which will hopefully put this question beyond doubt.

Patients with COPD want and deserve detailed assessment of their biology at the time of exacerbation to guide therapy. In a large multinational survey, patients with COPD insisted that they are willing to undergo detailed standardised physiological, imaging and biochemical testing at exacerbation and at follow-up [10]. Instead of assuming that less is more in COPD exacerbations, respiratory and family physicians need to lean into the challenge. International guidelines should be guided by these latest randomised controlled trial data [3–5, 9] and our patients' views [10], and argue for more intensive assessment and care for this morbid and deadly condition.
